# Nutrition, psychoneuroimmunology and depression: the therapeutic implications of omega-3 fatty acids in interferon-α-induced depression

**DOI:** 10.7603/s40681-015-0021-x

**Published:** 2015-11-28

**Authors:** Kuan-Pin Su

**Affiliations:** 1 Graduate Institute of Neural and Cognitive Sciences, School of Medicine, China Medical University, Taichung, Taiwan; 2 Department of Psychiatry and Mind-Body Research Center (MBI-Lab), China Medical University Hospital, 404 Taichung, Taiwan

**Keywords:** Inflammation, Depression, Omega-3 (n-3) polyunsaturated fatty acids (PUFAs), Interferon-α (IFN-α), Anti-inflammatory, Anti-depressant

## Abstract

The unmet need of current pharmacotherapy and the high occurrence of somatic symptoms and physical illness in depression imply that the ‘monoamine hypothesis’ is insufficient in approaching the aetiology of depression. Clinically, depressed patients manifest higher levels of inflammatory biomarkers, while proinflammatory cytokines induce neuropsychiatric symptoms (sickness behaviour) as well as major depressive episodes. Indeed, accumulating evidence suggests that inflammation dysregulation plays an important role in the pathophysiology of depression. Biological mechanisms that link inflammation to neuropsychiatric symptoms are vital in the understanding of the “mind-body” interface. IFN-α-induced depression is the most powerful support for the inflammation theory of depression. This clinical observation provides an excellent model for depression research. By comparing subjects with and without major depression induced by the cytokine treatment, statistical powers could be largely increased by reducing phenotypic variation (homogeneity in aetiological factors). In addition, the anti-inflammatory pathway has recently become an important topic in looking for new antidepressant therapies. For example, anti-inflammatory compounds, omega-3 polyunsaturated fatty acids (omega-3 PUFAs or n-3 PUFAs), have been found to be associated with the development and treatment for depression in human and animal models. Here I review recent epidemiological studies, cross-sectional and longitudinal case-controlled studies, interventional clinical trials, as well as basic animal and cellular studies to prove the linkage among omega-3 PUFAs, inflammation, and depression.

## 1. Introduction

Major depressive disorder (MDD) is a serious psychiatric illness with a high lifetime prevalence rate of up to one-tenth or possibly even one fifth [1]. Nevertheless, available treatments fail to meet the clinical needs of patients adequately, making this illness difficult to treat and burdensome to a patient’s life, family, and career. The growing burden of major depressive disorder (MDD) is evidenced by the projection that depression will become a leading cause of disease or injury worldwide by 2020 [2].

Clinical features, biological markers, and treatment outcomes for MDD are heterogeneous. Therefore, using our current diagnostic schemas undoubtedly contributes to the difficulty in finding any reliable biological markers for the disease [3]. According to the diagnostic criteria of the *Diagnostic and Statistical Manual of Mental Disorders*, 5th Edition (DSM-5), and the *International Statistical Classification of Diseases and Related Health Problems,* 10th Revision (ICD-10), individuals within the same diagnostic categories of MDD may have distinct clinical manifestations. Furthermore, the diagnostic classification does not provide reliable or predictive effects in treatment efficacy and/or the ability to predict the occurrence of adverse effects associated with specific antidepressants. Accordingly, with unsatisfactory outcomes for all the antidepressant treatments and the small-tomoderate effect sizes from all the biomarker studies and clinical trials, it is impossible to explain the whole picture of the aetiology of MDD with any single hypothesis.

**Fig. 1:**
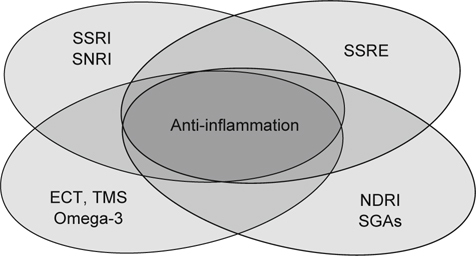
The current classification for antidepressant agents by monoamine reuptake mechanisms is insufficient to explain the aetiology of depression. The heterogeneity of depression could be reflected by the limits of pharmacotherapy and pharmacological classification based on serotonin, norepinephrine, and dopamine. Controversially, the agents that inhibit (*i.e.*, SSRI & SNRI), enhance (*i.e.*, SSRE), or even neglect (*i.e.*, NDRI & SGAs) the serotonin reuptake action could all be approved to be antidepressant treatments, yet all of them seem to share the common mechanism of antiinflammation. Interestingly, this common mechanisms is applicable not only for antidepressant agents from different categories but also for omega-3 PUFAs, electroconvulsive therapy (ECT) and repetitive transcranial magnetic stimulation (TMS).

The heterogeneity of depression could also be reflected by the current classification system with monoamine reuptake mechanisms for antidepressant agents (Figure 1). For example, the selective serotonin reuptake inhibitors (SSRIs) and serotoninnorepinephrine reuptake inhibitors (SNRIs), which inhibit serotonin reuptakes, are the most commonly prescribed antidepressant agents. However, tianepine, which enhances serotonin reuptakes, is also approved as an antidepressant treatment. Furthermore, we have antidepressant agents that have nothing to do with serotonin reuptakes, such as norepinephrine-dopamine reuptake inhibitors (NDRIs) and second-generation antipsychotics (SDA). Indeed, the limits of pharmacotherapy and pharmacological classification based on serotonin, norepinephrine, and dopamine imply that the ‘monoamine hypothesis’ is woefully insufficient in approaching the aetiology of depression. Interestingly, all the antidepressant treatments seem to share the common mechanism of anti-inflammation.

Inflammation theory lights a promising path to resolve the dilemma of depression. Administration of therapeutic cytokine interferon-α (IFN-α) can lead to clinical depression [4-6]. In fact, looking for antidepressant therapies from anti-inflammatory pathways has become a hot topic in current medical research [7]. Chronic inflammation is linked with early childhood trauma, major psychiatric disorders, and several physical diseases [8, 9]; inflammation theory thus provides a clear window to investigate mind-body interface.

Omega-3 polyunsaturated fatty acids (omega-3 PUFAs or n-3 PUFAs) are anti-inflammatory and have been proposed to be associated with the neurobiology of depression. The human body holds two main serial types of PUFAs: omega-6 (n-6) derived from cis-linoleic acid (LA, 18:2) and omega-3 (n-3) derived from α-linolenic acid (ALA, 18:3). Omega-3 PUFAs, eicosapentaenoic acid (EPA) and docosahexaenoic acid (DHA), and omega-6 PUFAs, arachidonic acid (AA), are important constituents of all cell membranes, essential for the survival of humans and other mammals [6, 10]. PUFAs appear active in biological functions; some of their functions require conversion to eicosanoids and products like prostaglandins (PGs), thromboxanes (TXs), and leukotrienes (LTs). A deficit of omega-3 PUFAs is reported to be associated with neurological, cardiovascular, cerebrovascular, autoimmune, and metabolic diseases, as well as bipolar disorder and depression [10]. This review summarizes current evidence about omega-3 PUFA biological mechanisms of and—inflammation in—depression.

## 2. Omega-3 fatty acids in depression

Societies with high consumption of fish in their diets appear to have a lower prevalence of MDD, mood disorders, coronary heart disease mortality, cardiovascular disease mortality, stroke mortality, and all-cause mortality [11], which implies a possibleprotective effect of omega-3 PUFAs in physical and psychiatric disorders. Consistent with epidemiological findings, patients with MDD show lower levels of omega-3 PUFAs in tissues of blood [12] and brain [13]. Deficits in omega-3 PUFA levels are reported in other populations with mood disorders: e.g., lower DHA and total omega-3 PUFAs in postpartum depression [14], lower DHA and EPA in social anxiety disorder [15], and lower DHA and AA in bipolar disorders [16].

Consistent with case-control studies of PUFA levels in human tissues, omega-3 PUFAs have been reported to be effective in treatment of MDD. Six meta-analytic reviews from four independent groups have reported on the antidepressant effect of PUFAs [17-22], yet three previous meta-analyses from the two of the four groups did not support these effects in heterogeneous populations (such as subclinical subjects in community samples) [23-26]. Negative findings must be interpreted with caution due to limitations: e.g., differing mood assessments, pooling heterogeneous populations, and implementing different intervention methods. For example, in a recent meta-analysis showing no benefits [24], their meta-analysis included clinical trials of enrolled individuals according to self-rating scales in settings like general practice surgery, a shopping mall and a university freshmen’s fair [27]. It found no beneficial effects of omega-3 PUFAs and was weighted 31.7% of a pooled estimate among a total of 13 clinical trials. Similar results emerged from the meta-analytic review by Appleton *et al.* [26]. Take one clinical trial [28] included in Appleton’s meta-analysis for example: Ness’s study enrolled a relatively large number of patients, 452, yet it did not focus on treating depression or using appropriate tools for the diagnosis of—as well as the severity rating of—depression. Intervention with omega-3 PUFAs was defined as “advising” subjects with angina to “eat more fish.” Treatment outcomes of omega-3 PUFAs in Ness’s study were negative and contributed greatly to the pooled estimate in Appleton’s meta-analysis.

Despite the uneven quality of published studies, recent metaanalytic evidence strongly supports the adjunctive use of omega-3 to treat bipolar depression [29]. However, studies regarding the effectiveness of omega-3 PUFAs in the acute manic phase of bipolar disorder are still lacking. To date, only one small doubleblind placebo-controlled trial has been published, and it does not support omega-3 PUFAs’ anti-manic effects [30, 31]. Omega-3 PUFAs offer promise in treating special populations with depression [32, 33]. Our 8-week double-blind, placebo-controlled study showed monotherapy with omega-3 PUFAs was associated with a significant improvement in depressive symptoms and a higher response rate in pregnant women with depression [34]. Most importantly, omega-3 PUFAs are safe for and well tolerated by depressed women during pregnancy and postpartum [35]. In addition, omega-3 PUFAs are proven effective and safe for children with depression [36], and supplementation with omega-3 PUFAs lowers the risk of suicide [37], alleviates MDD depressive symptoms associated with menopausal transition [38], and diminishes aggression in women with borderline personality disorder [39].

### 2.1. Safety and tolerability

Numerous clinical studies have shown omega-3 PUFAs are tolerated well by patients with chronic medical illnesses and mental disorders [6, 40-42]. Adverse reactions are rare; and if they occur at all, they usually involve belching, eructation or perhaps a fishy taste [43]. It is theorized that a potential anti-thrombotic effect of omega-3 PUFAs may increase the risk of bleeding. Clinical trials have shown that a high-dose consumption of omega-3 PUFAs is safe, even when concurrently administered with other agents that increase bleeding, such as aspirin and warfarin [40]. According to Harris’s systematic review on 19 available clinical trials with n-3 PUFAs supplementation for patients with an already high risk of bleeding (n = 4397) [44], the risk of clinically significant bleeding was virtually nonexistent. Another potential safety concern is the susceptibility of omega-3 fatty acids to undergo oxidation, which may contribute to patient intolerance and potential toxicity. Yet it must be said that conclusions on this issue are quite inconsistent [42]. Adding an antioxidant like vitamin E to omega-3 PUFAs is a common way to reduce oxidation and rancidity, maintain freshness, and increase shelf life. The concurrent use of vitamin E with omega-3 PUFAs may also overcome the potential risk of oxidative stress,yet most published studies show either unchanged or decreased oxidation [42]. Given omega-3 PUFAs’ antidepressant effects, another possible adverse effect is drug-induced mania. Yet until now, only one case report shows omega-3 PUFAs inducing hypomania [45]. A comprehensive assessment of manic symptoms in patients receiving omega-3 PUFAs is recommended for future clinical trials.

As depression is heterogeneous, any currently available antidepressant treatment only has modest effects. For example, the effect sizes of n-3 PUFAs in MDD treatment are only 0.17~0.23 [20, 22], which are similar to antidepressant drug treatments of 0.11 for mild to moderate, 0.17 for severe, and 0.47 for very severe MDD [46]. Therefore, it is of clinical interest to identify specific populations who might benefit from specific treatments.

## 3. Inflammation in depression

Accumulating evidence suggests that depression might be associated with activated inflammatory processes: e.g., depressed patients with elevated c-reactive protein (CRP), acute phase proteins, and pro-inflammatory cytokines [4, 7]. Depression is highly comorbid with chronic physical diseases [47]. In fact, children exposed to early-life adverse experiences display enduring low-grade systemic inflammation upon reaching adulthood [8], which is not only a risk factor for depression but also a feature of chronic physical diseases. Inflammation theory thus explains the high comorbidity of physical illness in depression and the potential “interface between mind and body [48].”

Systemic inflammatory challenges like lipopolysaccharide (LPS) or pro-inflammatory cytokine in experiments on animals cause behavioural changes induced by neuroinflammation that include anorexia, sleep abnormalities, reduction of locomotor activity and exploration, anhedonia, and cognitive disturbances, which share a strong similarity with the somatic symptoms of depression [4]. Sick individuals are often somewhat depressed and lethargic. The idea of a sickness’s behaviour emanates from a series of observed symptoms related to infection and cytokine/ prostaglandins administration in humans and animals. It offers a good model to study the effects of cytokine on the brain and behaviour [10, 49]. Excessive secretion of pro-inflammatory cytokines has been proposed to cause depression [50]. Microglia are the resident macrophages of the brain, and they act as the chief immune defense in the central nervous system (CNS) [51]. Upon activation, microglia up-regulate the expression of detrimental factors of reactive oxygen species such as nitric oxide via inducible nitric oxide synthase (iNOS) and induce oxidative stress, contributing to neuropsychiatric pathogenesis [52]. On the other hand, the expression of anti-oxidative enzymes like heme oxygenase-1 (HO-1) can reverse oxidative stress and may characterize antidepressant mechanisms [53, 54]. In addition, neuroinflammation reduces the survival of serotonergic neurons [55] and decreases neurogenesis [56], while antidepressants exert neuroprotection against microglia-mediated neurotoxicity [57].

### 3.1. IFN-α-induced depression

Substantiating evidence for the inflammation theory of depression is that interferon-alpha (IFN-α) induces clinical depression [5]. IFN-α is a standard cytokine therapy for chronic HCV infection, yet it associates with common and severe neuropsychiatric adverse effects. MDE during IFN-α therapy (IFN-α-induced depression) in patients with HCV is common, with incidence ranging from 23 to 45% [58]. Several biological mechanisms potentially play a role in this clinical phenomenon. For example, IFN-α-induced increases in IL-6 have been reported to predict development of depressive symptoms [59]. Cerebrospinal fluid (CSF) concentrations of 5-hydroxyindoleacetic acid (5-HIAA), but with no inflammatory markers, are associated with depressive symptoms induced by IFN-α [60]. Other studies are also mechanistically insightful by examining biomarkers such as plasma adrenocorticotropic hormone (ACTH), cortisol [61], serum tryptophan concentrations [62], and even brain function changes revealed in functional imagings [63]. Recent studies have identified genetic markers on serotonin transporters and interleukin-6 genes that seem to predict the development of IFN-α-induced depression [64].

## 4. Omega-3 fatty acids in interferon-α-induced depression

Chronic HCV infection is a major public health issue, and has a high rate of progression to liver cirrhosis and hepatocellular carcinoma [65, 66]. IFN-α is the standard therapy for chronic HCV infection, and will remain a cornerstone of therapy even with new combinations with ribavirin and protease inhibitors [67]. Because of the high rate of neuropsychiatric adverse effect like sickness behaviour and depression during IFN-α therapy, some clinicians consider adding prophylactic antidepressant use [68]. In patients with HCV infection, the prophylactic effects with SSRIs have been demonstrated by some [69-71], but not all [72-74] studies. Moreover, it has been associated with adverse events, including gastric discomfort, headache, dizziness, and an increased risk of rare but severe adverse effects, such as retinal haemorrhaging and cotton-wool spots [75, 76], bone marrow suppression, hepatotoxicity [74, 77], and manic episodes [78]. In addition, symptoms of IFN-α-induced sickness behaviour, once they develop, are only partially responsive to SSRIs [79]. As most patients receiving IFN-α do *not* develop clinically significant depression with IFN-a therapy, the routine pre-treatment with antidepressant drugs might expose patients to unnecessary medications. It is thus important to find alternative strategies for the prevention of IFN-α-induced depression.

One advantage of nutritional medicine is its application in early intervention that can avoid unnecessary exposure to medication. Omega-3 PUFAs have been shown in numerous clinical studies to be tolerated well by patients with chronic medical illnesses, including liver diseases (Mori, 2004; Bays, 2006; Mozaffarian and Rimm, 2006; Bays, 2007; Lee *et al.*, 2007). One of the hypothesized mechanisms underlying PUFAs’ antidepressant effects is their anti-inflammatory action [10]. Moreover, omega-3 PUFAs have been found to have beneficial effects in cytokineinduced behavioural changes in animal models of depression [80, 81]. Of particular relevance, our previous study demonstrated that lower omega-3 PUFA levels in the peripheral blood are associated with an increased risk of developing IFN-α-induced depression over the following weeks [5]. Based on this and the other evidence discussed above, we further conducted a 2-week, double-blind, placebo-controlled trial, to test the differential effects of the omega-3 PUFAs, EPA and DHA, against a placebo, in the prevention of IFN-α-induced depression. We have specifically prescribed a short (2 weeks) intervention *before* IFN-α therapy, in order to potentially correct the lower omega-3 fatty acid levels that we had previously identified as a risk factor for the development of IFN-α-induced depression [5]. According to most studies, the active antidepressant component from omega-3 PUFAs is EPA [20, 22], but we also wanted to test DHA because, as mentioned above, we have found that lower levels of this omega-3 PUFA predispose patients to IFN-induced depression [5].

The results of that newly published clinical trial [41] support our previous findings, showing that omega-3 PUFAs play a role in the risk of IFN-α-induced depression. To summarize, the incident rates of IFN-α-induced depression were significantly lower in EPA-, but not in DHA-treated patients (rates: 10% and 28%, respectively, *vs.* 30% for placebo, *P* = 0.037), as compared with the placebo. Both EPA and DHA pre-treatment significantly delayed the onset of IFN-induced depression (average weeks of onset: 12.0 and 11.7, respectively, vs. 5.3 for placebo, *P* = 0.002). Previous clinical trials and meta-analyses have shown that the efficacy of omega-3 fatty acids as antidepressants might be dependent on the selection of the subject populations as well as the ratio of EPA and DHA, and have further suggested that EPA, rather than DHA, might be the most active component of omega-3 PUFAs’ antidepressant effects [20, 22]. However, Mischoulon *et al.* found a dose-response effect supporting 1g/day as superior to 2 g/day or 4 g/day, though the latter study was limited by the lack of a placebo arm [82]. A recent meta-analysis has suggested that both EPA and DHA contribute to antidepressant effects, but that the effects of EPA are stronger [17]. Our current study, showing that EPA reduces the incidence of depression while DHA only delays the onset of depression, further supports this notion.

The anti-inflammatory action of omega-3 PUFAs is likely to be particularly important in the biological explanation for the antagonism of depressogenic effects of IFN-α. The model for IFN- α-induced depression reveals the increases of pro-inflammatory cytokines both in the periphery and in the brain of patients, with subsequent activation of the indoleamine 2,3-dioxygenase (IDO) pathway and the production of potentially depressogenic tryptophan metabolites, such as quinolinic acid [83]. EPA has numerous anti-inflammatory properties. Therefore, depressogenic mechanisms induced by proinflammatory cytokines and the IDO cascades are less likely to respond to standard antidepressants and more likely to respond to anti-inflammatory drugs [84-87]. In addition to this anti-inflammatory action, EPA and DHA may both exert their preventive effects also through neuroplasticity effects [88-90], which is a relevant molecular mechanism for antidepressant actions [91, 92].

## 5. Conclusions

The inflammation theory of depression draws support from several lines of evidence: e.g., increasing inflammatory biomarkers in clinical depression, and observed behavioral changes related to inflammatory activation. Interferon-α-induced depression in chronic HCV cases is the most notable clinical observation to support the inflammation theory of depression and an excellent model to probe the aetiology of depression in a prospective way. Anti-inflammatory omega-3 PUFAs prove beneficial in depression and several inflammation-related physical diseases. In addition, omega-3 PUFAs have been shown to have prophylactic effects in bipolar disorder [31, 42, 93, 94], psychotic transition in ultra-high risk individuals [95], and the development of posttraumatic stress disorder (PTSD) following accidental injury [96]. Furthermore, omega-3 PUFAs may particularly benefit children, pregnant women, and/or patients with comorbid cardiovascular or metabolic disorder, who all face greater risks of adverse effects from antidepressants, antipsychotics, and mood stabilizers. Therefore, our findings confirm and extend the notion that this nutritional intervention can have preventive effects in mental health populations, and they also corroborate the existing evidence that anti-inflammatory strategies may have antidepressant effects, especially in the context of depression associated with inflammation.

## Acknowledgments

Work included in this review was supported by the following grants: MOST103-2320-B-039-MY3, MOST103-2320-B-038-012-MY3, NSC 103-2923-B-039-002-MY3, 102-2911-I-039-501, 101-2628- B-039-001-MY3 and 101-2320-B-038-020-MY2 from the Ministry of Science and Techonology and CMU103-S-03, DMR-103- 078, 102-068 and 101-081 from the China Medical University in Taiwan.

## References

[1] Belmaker RH, Agam G. (2008). Major depressive disorder. N Engl J Med.

[2] Murray CJ, Lopez AD (1997). Alternative projections of mortality and disability by cause 1990-2020: Global Burden of Disease Study. Lancet.

[3] Hasler G, Drevets WC, Manji HK, Charney DS (2004). Discovering endophenotypes for major depression. Neuropsychopharmacology.

[4] Raison CL, Capuron L, Miller AH (2006). Cytokines sing the blues: inflammation and the pathogenesis of depression. Trends Immunol.

[5] Su KP (2010). Huang SY, Peng CY, Lai HC, Huang CL, Chen YC, *et al*. Phospholipase A2 and Cyclooxygenase 2 Genes Influence the Risk of Interferon-alpha-Induced Depression by Regulating Polyunsaturated Fatty Acids Levels. Biol Psychiatry.

[6] Su KP (2012). Inflammation in psychopathology of depression: Clinical, biological, and therapeutic implications. BioMedicine.

[7] Maes M, Leonard B, Fernandez A, Kubera M, Nowak G, Veerhuis R (2011). (Neuro)inflammation and neuroprogression as new pathways and drug targets in depression: from antioxidants to kinase inhibitors. Prog Neuropsychopharmacol Biol Psychiatry.

[8] Danese A, Moffitt TE, Pariante CM, Ambler A, Poulton R, Caspi A. (2008). Elevated inflammation levels in depressed adults with a history of childhood maltreatment. Arch Gen Psychiatry.

[9] Danese A, Pariante CM, Caspi A, Taylor A, Poulton R. (2007). Childhood maltreatment predicts adult inflammation in a life-course study. Proc Natl Acad Sci USA.

[10] Su KP (2009). Biological Mechanism of Antidepressant Effect of Omega-3 Fatty Acids: How Does Fish Oil Act as a ‘Mind-Body Interface’?. Neurosignals.

[11] Hibbeln JR, Nieminen LR, Blasbalg TL, Riggs JA, Lands WE (2006). Healthy intakes of n-3 and n-6 fatty acids: estimations considering worldwide diversity. Am J Clin Nutr.

[12] Lin PY, Huang SY, Su KP (2010). A meta-analytic review of polyunsaturated fatty acid compositions in patients with depression. Biol Psychiatry.

[13] McNamara RK, Hahn CG, Jandacek R, Rider T, Tso P, Stanford KE (2007). Selective deficits in the omega-3 fatty acid docosahexaenoic acid in the postmortem orbitofrontal cortex of patients with major depressive disorder. Biol Psychiatry.

[14] De Vriese SR, Christophe AB, Maes M. (2003). Lowered serum n-3 polyunsaturated fatty acid (PUFA) levels predict the occurrence of postpartum depression: further evidence that lowered n-PUFAs are related to major depression. Life Sci.

[15] Green P, Hermesh H, Monselise A, Marom S, Presburger G, Weizman A. (2006). Red cell membrane omega-3 fatty acids are decreased in nondepressed patients with social anxiety disorder. Eur Neuropsychopharmacol.

[16] Chiu CC, Huang SY, Su KP, Lu ML, Huang MC, Chen CC (2003). Polyunsaturated fatty acid deficit in patients with bipolar mania. Eur Neuropsychopharmacol.

[17] Sublette ME, Ellis SP, Geant AL, Mann JJ (2011). Meta-analysis of the effects of eicosapentaenoic acid (EPA) in clinical trials in depression. J Clin Psychiatry.

[18] Freeman MP, Mischoulon D, Tedeschini E, Goodness T, Cohen LS, Fava M (2010). Complementary and alternative medicine for major depressive disorder: a meta-analysis of patient characteristics, placebo-response rates, and treatment outcomes relative to standard antidepressants. J Clin Psychiatry.

[19] Freeman MP, Hibbeln JR, Wisner KL, Davis JM, Mischoulon D, Peet M (2006). Omega-3 fatty acids: evidence basis for treatment and future research in psychiatry. J Clin Psychiatry.

[20] Lin PY, Mischoulon D, Freeman MP, Matsuoka Y, Hibbeln J, Belmaker RH (2012). Are omega-3 fatty acids antidepressants or just mood-improving agents? The effect depends upon diagnosis, supplement preparation, and severity of depression. Mol Psychiatry.

[21] Lin PY, Su KP (2007). A meta-analytic review of double-blind, placebocontrolled trials of antidepressant efficacy of omega-3 fatty acids. J Clin Psychiatry.

[22] Martins JG, Bentsen H, Puri BK (2012). Eicosapentaenoic acid appears to be the key omega-3 fatty acid component associated with efficacy in major depressive disorder: a critique of Bloch and Hannestad and updated meta-analysis. Mol Psychiatry.

[23] Appleton KM, Hayward RC, Gunnell D, Peters TJ, Rogers PJ, Kessler D (2006). Effects of n-3 long-chain polyunsaturated fatty acids on depressed mood: systematic review of published trials. Am J Clin Nutr.

[24] Bloch MH, Hannestad J. (2012). Omega-3 fatty acids for the treatment of depression: systematic review and meta-analysis. Mol Psychiatry.

[25] Appleton KM, Rogers PJ, Ness AR (2008). Is there a role for n-3 longchain polyunsaturated fatty acids in the regulation of mood and behaviour? A review of the evidence to date from epidemiological studies, clinical studies and intervention trials. Nutr Res Rev.

[26] Appleton KM, Rogers PJ, Ness AR (2010). Updated systematic review and meta-analysis of the effects of n-3 long-chain polyunsaturated fatty acids on depressed mood. Am J Clin Nutr.

[27] Rogers PJ, Appleton KM, Kessler D, Peters TJ, Gunnell D, Hayward RC (2008). No effect of n-3 long-chain polyunsaturated fatty acid (EPA and DHA) supplementation on depressed mood and cognitive function: a randomised controlled trial. Br J Nutr.

[28] Ness AR, Gallacher JE, Bennett PD, Gunnell DJ, Rogers PJ, Kessler D (2003). Advice to eat fish and mood: a randomised controlled trial in men with angina. Nutr Neurosci.

[29] Sarris J, Mischoulon D, Schweitzer I. (2012). Omega-3 for bipolar disorder: meta-analyses of use in mania and bipolar depression. J Clin Psychiatry.

[30] Chiu CC, Huang SY, Chen CC, Su KP (2005). Omega-3 fatty acids are more beneficial in the depressive phase than in the manic phase in patients with bipolar I disorder. J Clin Psychiatry.

[31] Su KP (2000). Shen WW, Huang SY. Are omega3 fatty acids beneficial in depression but not mania?. Arch Gen Psychiatry.

[32] Su KP, Shen WW, Huang SY. The use of omega-3 fatty acids for the management of depression and psychosis during pregnancy and breast-feeding. In: Peet M, Glen I, Horrobin DF, editors. Phospholipid spectrum disorder in psychiatry and neurology. 2 ed. Carnforth: Marius Press; 2003. pp. 391–9.

[33] Chiu CC, Huang SY, Shen WW, Su KP. Omega-3 fatty acids for depression in pregnancy. Am J Psychiatry ; 160: 385. 10.1176/appi.ajp.160.2.385 12562593

[34] Su KP (2008). Huang SY, Chiu TH, Huang KC, Huang CL, Chang HC, *et al*. Omega-3 fatty acids for major depressive disorder during pregnancy: results from a randomized, double-blind, placebo-controlled trial. J Clin Psychiatry.

[35] Freeman MP (2006). Omega-3 fatty acids and perinatal depression: a review of the literature and recommendations for future research. Prostaglandins Leukot Essent Fatty Acids.

[36] Nemets H, Nemets B, Apter A, Bracha Z, Belmaker RH (2006). Omega-3 treatment of childhood depression: a controlled, double-blind pilot study. Am J Psychiatry.

[37] Hallahan B, Hibbeln JR, Davis JM, Garland MR (2007). Omega-3 fatty acid supplementation in patients with recurrent self-harm. Singlecentre double-blind randomised controlled trial. Br J Psychiatry.

[38] Freeman MP, Hibbeln JR, Silver M, Hirschberg AM, Wang B, Yule AM (2011). Omega-3 fatty acids for major depressive disorder associated with the menopausal transition: a preliminary open trial. Menopause.

[39] Zanarini MC, Frankenburg FR (2003). Omega-3 Fatty acid treatment of women with borderline personality disorder: a double-blind, placebo- controlled pilot study. Am J Psychiatry.

[40] Bays HE (2007). Safety considerations with omega-3 fatty acid therapy. Am J Cardiol.

[41] Su KP (2014). Lai HC, Yang HT, Su WP, Peng CY, Chang JP, *et al*. Omega-3 fatty acids in the prevention of interferon-alpha-induced depression: results from a randomized, controlled trial. Biol Psychiatry.

[42] Su KP, Wang SM, Pae CU (2013). Omega-3 polyunsaturated fatty acids for major depressive disorder. Expert Opin Investig Drugs.

[43] Bays H. Clinical overview of Omacor: a concentrated formulation of omega-3 polyunsaturated fatty acids. Am J Cardiol 2006; 98: 71i- 6i. 10.1016/j.amjcard.2005.12.029 16919519

[44] Harris WS (2007). Expert opinion: omega-3 fatty acids and bleeding-cause for concern?. Am J Cardiol.

[45] Kinrys G. (2000). Hypomania associated with omega-3 fatty acids. Arch Gen Psychiatry.

[46] Fournier JC, DeRubeis RJ, Hollon SD, Dimidjian S, Amsterdam JD, Shelton RC (2010). Antidepressant drug effects and depression severity: a patient-level meta-analysis. JAMA.

[47] Katon W, Sullivan MD. Depression and chronic medical illness. J Clin Psychiatry 1990; 51 Suppl: 3–11. 2189874

[48] Su KP (2008). Mind-body interface: the role of n-3 fatty acids in psychoneuroimmunology, somatic presentation, and medical illness comorbidity of depression. Asia Pac J Clin Nutr.

[49] Konsman JP, Parnet P, Dantzer R. (2002). Cytokine-induced sickness behaviour: mechanisms and implications. Trends Neurosci.

[50] Smith RS (1991). The macrophage theory of depression. Med Hypotheses.

[51] Hanisch UK, Kettenmann H. (2007). Microglia: active sensor and versatile effector cells in the normal and pathologic brain. Nat Neurosci.

[52] Lu DY, Leung YM, Su KP (2013). Interferon-alpha induces nitric oxide synthase expression and haem oxygenase-1 down-regulation in microglia: implications of cellular mechanism of IFN-alpha-induced depression. Int J Neuropsychopharmacol.

[53] Lu DY, Tsao YY, Leung YM, Su KP (2010). Docosahexaenoic acid suppresses neuroinflammatory responses and induces heme oxygenase-1 expression in BV-2 microglia: implications of antidepressant effects for omega-3 fatty acids. Neuropsychopharmacology.

[54] Gozzelino R, Jeney V, Soares MP (2010). Mechanisms of cell protection by heme oxygenase-1. Annu Rev Pharmacol Toxicol.

[55] Hochstrasser T, Ullrich C, Sperner-Unterweger B, Humpel C. (2011). Inflammatory stimuli reduce survival of serotonergic neurons and induce neuronal expression of indoleamine 2,3-dioxygenase in rat dorsal raphe nucleus organotypic brain slices. Neuroscience.

[56] Song C, Wang H. (2011). Cytokines mediated inflammation and decreased neurogenesis in animal models of depression. Prog Neuropsychopharmacol Biol Psychiatry.

[57] Zhang F, Zhou H, Wilson BC, Shi JS, Hong JS, Gao HM (2012). Fluoxetine protects neurons against microglial activation-mediated neurotoxicity. Parkinsonism Relat Disord.

[58] Asnis GM, De La GR (2006). Interferon-induced depression in chronic hepatitis C: a review of its prevalence, risk factors, biology, and treatment approaches. J Clin Gastroenterol.

[59] Bonaccorso S, Puzella A, Marino V, Pasquini M, Biondi M, Artini M (2001). Immunotherapy with interferon-alpha in patients affected by chronic hepatitis C induces an intercorrelated stimulation of the cytokine network and an increase in depressive and anxiety symptoms. Psychiatry Res.

[60] Raison CL, Borisov AS, Majer M, Drake DF, Pagnoni G, Woolwine BJ (2009). Activation of central nervous system inflammatory pathways by interferon-alpha: relationship to monoamines and depression. Biol Psychiatry.

[61] Capuron L, Raison CL, Musselman DL, Lawson DH, Nemeroff CB, Miller AH (2003). Association of exaggerated HPA axis response to the initial injection of interferon-alpha with development of depression during interferon-alpha therapy. Am J Psychiatry.

[62] Capuron L, Neurauter G, Musselman DL, Lawson DH, Nemeroff CB, Fuchs D (2003). Interferon-alpha-induced changes in tryptophan metabolism. relationship to depression and paroxetine treatment. Biol Psychiatry.

[63] Capuron L, Pagnoni G, Demetrashvili M, Woolwine BJ, Nemeroff CB, Berns GS (2005). Anterior cingulate activation and error processing during interferon-alpha treatment. Biol Psychiatry.

[64] Bull SJ, Huezo-Diaz P, Binder EB, Cubells JF, Ranjith G, Maddock C (2009). Functional polymorphisms in the interleukin-6 and serotonin transporter genes, and depression and fatigue induced by interferonalpha and ribavirin treatment. Mol Psychiatry.

[65] Lauer GM, Walker BD (2001). Hepatitis C virus infection. N Engl J Med.

[66] Poynard T, Yuen MF, Ratziu V, Lai CL (2003). Viral hepatitis C. Lancet.

[67] Schaefer M, Capuron L, Friebe A, ez-Quevedo C, Robaeys G, Neri S (2012). Hepatitis C infection, antiviral treatment and mental health: a European expert consensus statement. J Hepatol.

[68] Dieperink E, Ho SB, Thuras P, Willenbring ML (2003). A prospective study of neuropsychiatric symptoms associated with interferon-alpha-2b and ribavirin therapy for patients with chronic hepatitis C. Psychosomatics.

[69] Raison CL, Woolwine BJ, Demetrashvili MF, Borisov AS, Weinreib R, Staab JP (2007). Paroxetine for prevention of depressive symptoms induced by interferon-alpha and ribavirin for hepatitis C. Aliment Pharmacol Ther.

[70] de Knegt RJ, Bezemer G, Van Gool AR, Drenth JP, Hansen BE, Droogleever Fortuyn HA (2011). Randomised clinical trial: escitalopram for the prevention of psychiatric adverse events during treatment with peginterferon-alfa-2a and ribavirin for chronic hepatitis C. Aliment Pharmacol Ther.

[71] Schaefer M, Sarkar R, Knop V, Effenberger S, Friebe A, Heinze L (2012). Escitalopram for the Prevention of Peginterferon-alpha2a-Associated Depression in Hepatitis C Virus-Infected Patients Without Previous Psychiatric Disease: A Randomized Trial. Ann Intern Med.

[72] Diez-Quevedo C, Masnou H, Planas R, Castellvi P, Gimenez D, Morillas RM (2011). Prophylactic treatment with escitalopram of pegylated interferon alfa-2a-induced depression in hepatitis C: a 12- week, randomized, double-blind, placebo-controlled trial. J Clin Psychiatry.

[73] Morasco BJ, Loftis JM, Indest DW, Ruimy S, Davison JW, Felker B (2010). Prophylactic antidepressant treatment in patients with hepatitis C on antiviral therapy: a double-blind, placebo-controlled trial. Psychosomatics.

[74] Morasco BJ, Rifai MA, Loftis JM, Indest DW, Moles JK, Hauser P. (2007). A randomized trial of paroxetine to prevent interferon-alpha-induced depression in patients with hepatitis C. J Affect Disord.

[75] Hejny C, Sternberg P, Lawson DH, Greiner K, Aaberg TM (2001). Retinopathy associated with high-dose interferon alfa-2b therapy. Am J Ophthalmol.

[76] Musselman DL, Lawson DH, Gumnick JF, Manatunga AK, Penna S, Goodkin RS (2001). Paroxetine for the prevention of depression induced by high-dose interferon alfa. N Engl J Med.

[77] Loftis JM, Hauser P. (2003). Safety of the treatment of interferon-alphainduced depression. Psychosomatics.

[78] Wu PL, Liao KF, Peng CY, Pariante CM, Su KP (2007). Manic episode associated with citalopram therapy for interferon-induced depression in a patient with chronic hepatitis C infection. Gen Hosp Psychiatry.

[79] Capuron L, Gumnick JF, Musselman DL, Lawson DH, Reemsnyder A, Nemeroff CB (2002). Neurobehavioral effects of interferon-alpha in cancer patients: phenomenology and paroxetine responsiveness of symptom dimensions. Neuropsychopharmacology.

[80] Song C, Phillips AG, Leonard BE, Horrobin DF (2004). Ethyl-eicosapentaenoic acid ingestion prevents corticosterone-mediated memory impairment induced by central administration of interleukin-1beta in rats. Mol Psychiatry.

[81] Song C, Leonard BE, Horrobin DF (2004). Dietary ethyl-eicosapentaenoic acid but not soybean oil reverses central interleukin-1-induced changes in behavior, corticosterone and immune response in rats. Stress.

[82] Mischoulon D, Best-Popescu C, Laposata M, Merens W, Murakami JL, Wu SL (2008). A double-blind dose-finding pilot study of docosahexaenoic acid (DHA) for major depressive disorder. Eur Neuropsychopharmacol.

[83] Raison CL, Dantzer R, Kelley KW, Lawson MA, Woolwine BJ, Vogt G (2010). CSF concentrations of brain tryptophan and kynurenines during immune stimulation with IFN-alpha: relationship to CNS immune responses and depression. Mol Psychiatry.

[84] Cattaneo A, Gennarelli M, Uher R, Breen G, Farmer A, Aitchison KJ (2013). Candidate genes expression profile associated with antidepressants response in the GENDEP study: differentiating between baseline ‘predictors’ and longitudinal ‘targets’. Neuropsychopharmacology.

[85] Carvalho LA, Torre JP, Papadopoulos AS, Poon L, Juruena MF, Markopoulou K (2013). Lack of clinical therapeutic benefit of antidepressants is associated overall activation of the inflammatory system. J Affect Disord.

[86] Zunszain PA, Anacker C, Cattaneo A, Choudhury S, Musaelyan K, Myint AM (2012). Interleukin-1beta: a new regulator of the kynurenine pathway affecting human hippocampal neurogenesis. Neuropsychopharmacology.

[87] Raison CL, Rutherford RE, Woolwine BJ, Shuo C, Schettler P, Drake DF (2013). A randomized controlled trial of the tumor necrosis factor antagonist infliximab for treatment-resistant depression: the role of baseline inflammatory biomarkers. JAMA Psychiatry.

[88] Bazinet RP, Laye S. (2014). Polyunsaturated fatty acids and their metabolites in brain function and disease. Nat Rev Neurosci.

[89] Rao JS, Ertley RN, Lee HJ, DeMar JC, Arnold JT, Rapoport SI (2007). n-3 polyunsaturated fatty acid deprivation in rats decreases frontal cortex BDNF via a p38 MAPK-dependent mechanism. Mol Psychiatry.

[90] Beltz BS, Tlusty MF, Benton JL, Sandeman DC (2007). Omega-3 fatty acids upregulate adult neurogenesis. NeurosciLett.

[91] Castren E, Hen R. Neuronal plasticity and antidepressant actions. Trends Neurosci 2013. 10.1016/j.tins.2012.12.010 PMC3648595 23380665

[92] Eisch AJ, Petrik D. (2012). Depression and hippocampal neurogenesis: a road to remission?. Science.

[93] Stoll AL, Severus WE, Freeman MP, Rueter S, Zboyan HA, Diamond E (1999). Omega 3 fatty acids in bipolar disorder: a preliminary double-blind, placebo-controlled trial. Arch Gen Psychiatry.

[94] Su KP, Balanzá-Martínez V., McNamara RK (2013). Role of omega-3 fatty acids in mood disorders. The omega-3 fatty acid deficiency syndrome: opportunities for disease prevention.

[95] Amminger GP, Schafer MR, Papageorgiou K, Klier CM, Cotton SM, Harrigan SM (2010). Long-chain omega-3 fatty acids for indicated prevention of psychotic disorders: a randomized, placebo-controlled trial. Arch Gen Psychiatry.

[96] Matsuoka Y, Nishi D, Yonemoto N, Hamazaki K, Hashimoto K, Hamazaki T. (2010). Omega-3 fatty acids for secondary prevention of posttraumatic stress disorder after accidental injury: an open-label pilot study. J Clin Psychopharmacol.

